# Characterisation of Cellulose Synthase Like F6 (*CslF6*) Mutants Shows Altered Carbon Metabolism in β-D-(1,3;1,4)-Glucan Deficient Grain in *Brachypodium distachyon*

**DOI:** 10.3389/fpls.2020.602850

**Published:** 2021-01-11

**Authors:** Melissa Bain, Allison van de Meene, Rafael Costa, Monika S. Doblin

**Affiliations:** ^1^Australian Research Council (ARC) Centre of Excellence in Plant Cell Walls, The School of BioSciences, The University of Melbourne, Parkville, VIC, Australia; ^2^Institute of Plant Sciences Paris-Saclay (IPS2), Centre National de la Recherche Scientifique (CNRS), L’Institut National de Recherche pour L’Agriculture, L’Alimentation et L’Environnement (INRAE), Univ Evry, Université Paris-Saclay, Orsay, France; ^3^Centre National de la Recherche Scientifique (CNRS), L’Institut National de Recherche pour L’Agriculture, L’Alimentation et L’Environnement (INRAE), Institute of Plant Sciences Paris-Saclay (IPS2), Université de Paris, Orsay, France; ^4^Department of Animal Plant and Soil Sciences, La Trobe Institute for Agriculture and Food (LIAF), La Trobe University, Melbourne, VIC, Australia

**Keywords:** *Brachypodium distachyon*, β-D-(1, 3;1, 4)-glucan, endosperm, cell wall, starch

## Abstract

*Brachypodium distachyon* is a small, fast growing grass species in the *Pooideae* subfamily that has become established as a model for other temperate cereals of agricultural significance, such as barley (*Hordeum vulgare*) and wheat (*Triticum aestivum*). The unusually high content in whole grains of β-D-(1,3;1,4)-glucan or mixed linkage glucan (MLG), considered a valuable dietary fibre due to its increased solubility in water compared with cellulose, makes *B. distachyon* an attractive model for these polysaccharides. The carbohydrate composition of grain in *B. distachyon* is interesting not only in understanding the synthesis of MLG, but more broadly in the mechanism(s) of carbon partitioning in cereal grains. Several mutants in the major MLG synthase, cellulose synthase like (CSL) F6, were identified in a screen of a TILLING population that show a loss of function *in vitro*. Surprisingly, loss of *cslf6* synthase capacity appears to have a severe impact on survival, growth, and development in *B. distachyon* in contrast to equivalent mutants in barley and rice. One mutant, A656T, which showed milder growth impacts in heterozygotes shows a 21% (w/w) reduction in average grain MLG and more than doubling of starch compared with wildtype. The endosperm architecture of grains with the A656T mutation is altered, with a reduction in wall thickness and increased deposition of starch in larger granules than typical of wildtype *B. distachyon*. Together these changes demonstrate an alteration in the carbon storage of *cslf6* mutant grains in response to reduced MLG synthase capacity and a possible cross-regulation with starch synthesis which should be a focus in future work in composition of these grains. The consequences of these findings for the use of *B. distachyon* as a model species for understanding MLG synthesis, and more broadly the implications for improving the nutritional value of cereal grains through alteration of soluble dietary fibre content are discussed.

## Introduction

*Brachypodium distachyon*, a fast growing grass of small stature with a small diploid (271.9 Mbp) sequenced genome (The International Brachypodium Initiative, 2010), has emerged as a model species for other temperate grasses of agricultural importance. A member of the *Pooideae* subfamily, *B. distachyon* is more closely related to members of the *Triticeae* (wheat, barley, rye), *Aveneae* (oat), and *Poeae* (*Lolium* spp.) tribes than other common grass models including rice (*Oryza sativa*), *Sorghum bicolor* and maize (*Zea mays*) (Catalán et al., 1997; Grass Phylogeny Working Group et al., 2001; Kellogg, 2001; The International Brachypodium Initiative, 2010; Hands and Drea, 2012; Hochbach et al., 2015). Thus the number of genetic and genomic resources for *B. distachyon* is increasing rapidly and includes Targeted Induced Local Lesions IN Genomes (TILLING) (Dalmais et al., 2013) and TDNA collections (Bragg et al., 2012; Hsia et al., 2017), as well as transcript maps and comparative co-expression network analysis tools (Sibout et al., 2017).

*B. distachyon* has been shown to have a similar grain structure compared to other temperate grasses, in addition to developmental characteristics (Guillon et al., 2011; Opanowicz et al., 2011) which are also similar to wheat, barley, rice, and maize (Hands and Drea, 2012; Trafford et al., 2013). However, the partitioning of carbon resources into storage carbohydrates in the endosperm is a key point of difference between *B. distachyon* and other *Pooideae* (Hands and Drea, 2012). Whilst the cell walls of aerial tissues of seedlings are very similar between *B. distachyon*, wheat, and barley (Christensen et al., 2010), the endosperm walls of *B. distachyon* grain are thicker with greater deposition of polysaccharides (Guillon et al., 2011; Opanowicz et al., 2011; Trafford et al., 2013). This is largely due to the amount of the polysaccharide β-D-(1,3;1,4)-glucan or mixed linkage glucan (MLG), at much higher amounts per grain weight, upwards of four times that of close relatives including barley and wheat (Guillon et al., 2011; Opanowicz et al., 2011; Hands and Drea, 2012; Trafford et al., 2013). In most grains of domesticated cereals, the starch content has been bred to be up to 70% of grain weight, in contrast to typically less than 10% MLG (w/w) (Trafford et al., 2013). However, the converse is true for *B. distachyon* where most carbohydrate is in MLG, up to 45% (w/w), and the starch content is only 6% of grain weight (Guillon et al., 2011), suggestive of a role for MLG as an alternative carbon sink of readily hydrolysed sugars available to germinating grain (Guillon et al., 2011; Burton and Fincher, 2012; Trafford et al., 2013).

A homopolymer of glucose (Glc), MLG has a unique structure comprising stretches of β-(1,4)-linkages interspersed with β-(1,3)-linkages, the arrangement of which confer its increased solubility compared with cellulose (Burton and Fincher, 2009; Fincher, 2009b; Collins et al., 2010). In cereals, most MLG is arranged in stretches of either two or three β-(1,4)-linked residues between single β-(1,3)-linkages, such that the structure consists predominantly of cellotriosyl (degree of polymerisation 3; DP3) and cellotetraosyl (DP4) units, although longer stretches are also present (Staudte et al., 1983; Woodward et al., 1983). Due to its unique properties and abundance in some cereal grains MLG is an important source of soluble dietary fibre and has been linked with human health benefits such as reduced risk of type II diabetes, colorectal cancer, and cardiovascular disease (Collins et al., 2010).

**FIGURE 1:**
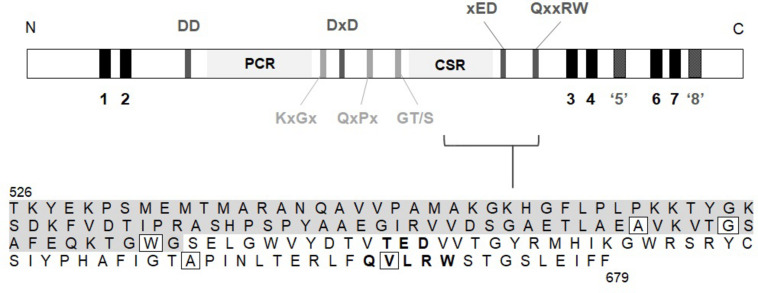
Region within the catalytic domain of *Bd*CSLF6 which was screened for mutations by NGS in the TILLING population. A schematic of the protein sequence includes; predicted transmembrane helices (TMH) (black); the characteristic catalytic domain DD, DxD, xED, and QxxRW motifs (dark grey); plant conserved region (PCR) and class-specific region (CSR); and key conserved structural motifs (light grey). TMH5 is shown in dark grey and inverted commas as it is predicted by modelling to lie adjacent to, rather than traversing, the membrane based on homology to interfacial helix (IF) 3 in the BcsA crystal structure (Morgan et al., 2013) (model of *Bd*CSLF6 shown in Figure 4). Due to lack of homology to BcsA, and protease protection assays showing topology such that the N- and C-termini are both cytosolic (Kim et al., 2015), TMH8 is also not predicted to traverse the membrane (dark grey, inverted commas). The amino acid sequence of the target region is shown, from residue T526 to F679, residues within the CSR (Sethaphong et al., 2013; Dimitroff et al., 2016) indicated with grey shading and the xED and QxxRW motifs in bold. Boxed residues signify mutations identified in the screen that give rise to changes in the amino acid sequence.

MLG is synthesised by members of the Glycosyltransferase (GT) 2 family (Coutinho et al., 2003) of uridine diphosphate glucose (UDP-Glc) utilising enzymes^1^ (Lombard et al., 2014). The genes responsible for MLG synthesis in grasses share sequence homology to the cellulose synthases (*CesAs*), and belong to the cellulose synthase like (*Csl*) *F, H*, and *J* sub-families (Burton et al., 2006; Farrokhi et al., 2006; Doblin et al., 2009; Little et al., 2018). *CslF6* is the major isoform contributing to total MLG in both vegetative and floral tissues in barley (Burton et al., 2008; Tonooka et al., 2009; Taketa et al., 2012), wheat (Nemeth et al., 2010), rice (Vega-Sánchez et al., 2012) and *B. distachyon* (Trafford et al., 2013). The CSLF, H and J proteins are characterised by a large cytosolic catalytic domain containing the signature GT2 motifs, DD, DxD, xED, QxxRW, including the plant conserved (PCR) and class-specific (CSR) regions, and are integral membrane proteins (Saxena et al., 1995; Charnock and Davies, 1999; Figure 1).

Several mutants of *CslF6* have been characterised which lead to reductions in MLG content in barley (Tonooka et al., 2009; Cory et al., 2012; Taketa et al., 2012; Hu et al., 2014; Wong et al., 2015), rice (Vega-Sánchez et al., 2012), and wheat (Nemeth et al., 2010), and were reasonably well tolerated, resulting in moderate reductions in vegetative growth. In wheat targeted RNAi knockdowns of *CslF6* driven by an endosperm-specific promoter were able to reduce the MLG content of whole grain flour by 30–53% (Nemeth et al., 2010). Induced single nucleotide polymorphisms (SNPs) in barley “beta glucanless *(bgl)*” lines (Tonooka et al., 2009) were found to contain mutations in the PCR domain as well as between the xED and QxxRW motifs, resulting in undetectable levels of MLG compared with 3.2% (w/w) in wildtype grain (Taketa et al., 2012). No detectable MLG was reported in either vegetative or floral tissues of *bgl* mutants, with plants showing a 30% reduction in mature height, some chlorosis of leaves and a slight decrease in yield but normal germination rates (Tonooka et al., 2009; Taketa et al., 2012). Similarly, TDNA knockout mutants of *CslF6* in rice were also able to tolerate a reduction of MLG of 97% in coleoptiles, and to undetectable levels in other tissues, with normal development aside from a 33% reduction in height (Vega-Sánchez et al., 2012).

Given that grain composition is central to the nutritional value of cereals in human diets, understanding how the endosperm develops is a focus in cereal research. Alterations in the composition of barley grain away from high starch content toward increased amounts of non-starch polysaccharides, including β-glucans (Bird et al., 2004a, b), have previously been shown to translate to increases in markers of general bowel health (Bird et al., 2008; McOrist et al., 2011) and reductions in blood insulin (King et al., 2008) when included in human diets. The distinct composition of *B. distachyon* endosperm which is high in MLG and low in starch therefore makes it an ideal candidate species for understanding the biosynthesis and regulation of this wall polysaccharide and the implications this may have for the effective manipulation of the nutritional value of commercially important cereal grains. *B. distachyon* is also an attractive model species for prospective *in vitro* work to characterise MLG biosynthesis in a native context because it can be easily transformed and requires less time and resources to complete an entire life cycle than species such as barley. A reduced or null MLG line in *B. distachyon* genetically complemented with a fluorescently tagged version of CSLF6, for example, would facilitate experiments targeting fundamental questions about the mechanism of MLG biosynthesis by facilitating dynamic protein localisation studies to target sites of synthesis, deposition, and turnover during development.

The work presented here seeks to establish a *cslf6* loss of function mutant in *B. distachyon* to provide a readily transformable, low or no MLG background for further studies of the synthesis of this polysaccharide in a native context. We present our assessment of several *cslf6* mutations discovered amongst a TILLING population, and the surprising observation of a severe impact of loss of MLG synthase capacity on growth and development in *B. distachyon*. To investigate the likely consequences of loss of MLG synthase capacity in grain, we show increased starch content and changes to endosperm architecture in a mutant which displays loss of function *in vitro*, indicating that the regulation of these two polysaccharides may be linked. The viability of *B. distachyon* as a model for investigation of MLG biosynthesis and regulation and the implications of this altered carbon metabolism for future research into the soluble fibre composition of these grains are discussed.

## Materials and Methods

### High Throughput Screen of *B. distachyon* TILLING Population

The M1 generation of the TILLING population of inbred line Bd21-3 (Dalmais et al., 2013) was screened at the INRAE-IPS2 TILLING platform by next generation sequencing (NGS) for mutations in a target region of the Bradi3g16307.1 CDS from nucleotide 1,575 to 2,037 (Figure 1). The target region was amplified using the following primers with adapters for paired-end HiSeq (Illumina) sequencing underlined; For 5′ TTCCCTACACGACGCTCTTCCGATCTGACCAAGTATGAG AAGCCCA 3′; and Rev 5′ AGTTCAGACGTGTGCTCTTC CGATCTGAAGAAGATCTCGAGGGAGC 3′. The M2 progeny for sequence-verified SNPs were obtained.

### Growth Conditions for *B. distachyon* Lines

Grains were imbibed in water on Whatman 3MM paper overnight and planted individually in 20 cm pots in commercial seed raising mix (Debco) at 1 cm below the surface. Plants were then grown at 21°C under an 8 h dark and 16 h light cycle in controlled environment Thermoline growth cabinets with fluorescent white light for the duration of the experiment.

Growth was monitored across 100 days and entry into key developmental stages, along a modified BBCH (Biologische Bundesantalt, Bundessortenamt, and Chemische Industrie) scale developed for *B. distachyon* by Hong et al. (2011) was recorded for each individual.

### Genotyping of TILLING Lines

Leaf material was harvested from juvenile plants for gDNA extraction and ethanol precipitation as described by Edwards et al. (1991). Individuals were genotyped using derived cleaved amplified polymorphic sequence (dCAPS) markers designed using dCAPS Finder 2.0^2^ (Neff et al., 2002). Primers introduce restriction sites in the presence of the mutations (Supplementary Table 1), and genotyping was validated by sequencing.

### Generation of Wildtype and Mutant *BdCslF6* Expression Constructs

The wildtype coding sequence of *BdCslF6* was amplified from cDNA derived from leaves of Bd21-1 2 weeks old seedlings and cloned under the CaMV 35S promoter into a modified pGreenII vector (Hellens et al., 2000), which included an octopine synthase (OCS) terminator sequence (Wilson et al., 2015), using the Gibson Assembly Cloning Kit (NEB). Each TILLING variant was generated from this construct by introducing the SNP into the primer sequences (Supplementary Table 2), and verified by sequencing.

### Heterologous Expression of *BdCslF6* Variants in *N. benthamiana*

Constructs were co-transformed with the pSOUP helper plasmid (Hellens et al., 2000) into *Agrobacterium tumefaciens* strain AGL1, and grown for 2 days in 2YT. Cells were collected by centrifugation, resuspended to a final OD_600_ 0.6–0.8 with 10 mM MgCl_2_ and 0.8 mM acetosyringone, and incubated for 2 h at room temperature. Constructs were co-infiltrated into triplicate leaves of *Nicotiana benthamiana* of equivalent ages with a culture carrying the P19 suppressor of gene silencing (Voinnet et al., 2003) as described in Wilson et al. (2015). Transformed leaves were collected 2 days post-infiltration (DPI) and snap-frozen in liquid nitrogen for subsequent analysis.

### Extraction of Protein and Analysis by Immunoblotting

Leaves were ground in a mortar and pestle in 50 mM potassium phosphate buffer pH 7.5, 20 mM potassium chloride, 200 mM sucrose, 0.2 mM PMSF, 10 mM DTT and 1 × cOmplete protease inhibitor cocktail (Roche). Homogenate was filtered through Miracloth (Merck), centrifuged at 10,000×*g* for 10 min at 4°C and the resulting pellet processed for cell wall material. Microsomal membranes (MM) were collected from this low speed supernatant after centrifugation at 100,000×*g* for 60 min at 4°C, and resuspended in homogenising buffer without DTT. Immunoblotting was performed as described by Wilson et al. (2015) with membrane processing as described by Doblin et al. (2009). CSLF6 was detected using either a polyclonal antibody (1:2,000 dilution) generated against the peptide AKGKHGFLPLPKKTYGK (Wilson et al., 2015), or a monoclonal antibody (1:500) derived from a hybridoma line (7G3A6) generated against the same peptide (Y.Y. Ho, unpublished). Immunoblots were scanned using a Chemidoc MP Imager (BioRad) and signal intensity of bands observed measured using Image Lab software (BioRad).

### Preparation of Cell Walls and Quantification of MLG

Cell walls were prepared as alcohol insoluble residues (AIR) from the 10,000×*g* pellets from MM preparations as described in Pettolino et al. (2012). For MLG quantification, AIR was resuspended to 20 mg/mL in 20 mM sodium phosphate buffer pH 6.5, swollen at 90°C for 20 min, cooled and then digested with 2 U lichenase (Megazyme) for 1.5 h at 50°C whilst shaking at 750 rpm. Reactions were stopped with one volume of 200 mM sodium acetate, pH 4.0. Undigested wall was removed at 8,000×*g* for 10 min, and polysaccharides precipitated with four volumes of ethanol for 2 h at −20°C before removal at 15,000×*g* for 10 min. The resulting supernatant containing the released oligosaccharides was dried under nitrogen and resuspended in 200 μL ultrapure water. Oligosaccharides were separated on a Dionex LC (Thermo Fisher Scientific) by high performance anion exchange chromatography (HPAEC), detected using pulsed ampherometric detection (PAD) as described by Doblin et al. (2009), and quantified from peak area (nC min) obtained using Chromeleon 6.8 Chromatography Data System software (Thermo Fisher Scientific) against digested barley flour MLG standard (Megazyme).

To compare content of leaves transformed with CSLF6 variant constructs, MLG was normalised to corresponding *in vitro* protein on immunoblot, relative to a *Bd*CSLF6 wildtype control on each, and analysed by analysis of variance (ANOVA) using R v 3.3.0 (R Core Team, 2016).

### Assay for MLG and Starch Content of Grains

To allow for the estimation of both MLG and starch on equivalent material, both assays were performed on tissue pooled from five randomly selected M6 grains from each line. Grains were ground thoroughly to a fine powder using a Qiagen Tissue Lyser II with a 3 mm tungsten carbide bead (Qiagen). Homogenised pooled tissue was weighed into four replicates of 2–4 mg, and two β-glucan (MLG; Megazyme) and two total starch assays (Megazyme) performed, and results averaged.

### Transcript Analysis by qRT-PCR

For each line 4–6 developing grains with the husk manually removed were collected from different individuals 8 to 10 days after pollination (DAP), embryos removed and individually snap frozen in liquid nitrogen. After grinding frozen grain with a pestle, total RNA was extracted with the RNeasy mini kit (Qiagen), treated with 1 U DNase I (Invitrogen) and cDNA synthesised from 500 μg with 200 U Superscript III (Invitrogen) using an oligo dT_15_ primer.

Absolute transcript quantitation was performed in triplicate using standards for *CslF* and *H* family genes provided by Dr Neil Shirley (The University of Adelaide, Australia) as described by Trafford et al. (2013), including the *CslF10-2* (Bradig25157.1) gene, a paralogue of *CslF10-1* (Bradig25150.1) (Ermawar et al., 2015). Analyses were performed on a CFX384 Touch Real-Time PCR system (BioRad) using KAPA Fast qRT-PCR chemistry. Quantitation against standards was performed using CFX Maestro Analysis software (BioRad), and normalisation of gene expression calculated using the GeNORM method as described by Vandesompele et al. (2002). Normalised transcript abundance was analysed with two factor ANOVA with pairwise comparisons using R (R Core Team, 2016).

### Confocal Microscopy of Grains

At least three biological replicates of mature grains were sampled at 18–20 DAP from each line. Grains were cut into 2–3 mm cross sections and fixed in 4% (v/v) paraformaldehyde and 0.5% (v/v) glutaraldehyde overnight at 4°C. Grains were then dehydrated in an ethanol series using stepwise washes of 10% (v/v) to 100% prior to infiltration with LRW resin (ProSciTech) before embedding and preparation of 1 μm transverse sections. For Toluidine Blue O (TBO) staining, dried sections were incubated in 0.01% (v/v) TBO for 30 s and washed in distilled water before being mounted in DPX solution (Merck) for imaging. Lugol solution (Sigma) for starch or Pontamine Fast Scarlet 4B (S4B; Sigma Aldrich) stain for cellulose was added directly to sections for 60 s then washed off with distilled water, and sections mounted in PBS for immediate imaging. Sections were prepared for immunolabelling by blocking in 3% (w/v) BSA before incubation in α-MLG (1:200; BG1, Biosupplies) or α-xylan LM10 or LM11 (Plant Probes), for 1 h at room temperature then 4°C overnight. After washing three times in PBS for 5 min each time, sections were incubated in Alexa Fluor 586 α-mouse (1:100, Abcam) for 2 h at room temperature, washed again, and mounted in Citifluor antifadent (EMS) for imaging.

Images were acquired using a Leica DM6000 compound microscope with DFC 300 CCD camera (Leica Microsystems), and MetaMorph acquisition software. Cell numbers and areas were analysed with FIJI software (NIH). Briefly, the cell walls in the endosperm were selected using grey scale threshold and a binary image was then created. The number and areas of cells were calculated using the Analyse Particles command. To measure the thickness of the cell walls, a transect line was drawn from the nucellar projection to the opposite surface of the grain (Trafford et al., 2013) and measured using a plot profile in FIJI.

### Transmission Electron Microscopy (TEM) of Stems

Tissue was collected from each of the *B. distachyon* lines from the first internode of elongating stems at 3 weeks post germination and cryofixed using a Leica EMPACT2 high pressure freezer (Leica Microsystems). Following fixation the samples were freeze substituted using 0.2% uranyl acetate in acetone for 72 h at −85°C before being gradually brought to room temperature over 24 h. The samples were washed once in acetone followed by three washes in ethanol. Infiltration with LRW resin (ProScitech) was performed in a dilution series of 25, 50, 75% LRW:ethanol, followed by three incubations in 100% LRW for 8 h at each step. The samples were embedded in gelatin capsules and polymerised at 55°C for 24 h. Thin sections (70 nm) were cut using a Leica UC7 ultramicrotome (Leica Microsystems) and collected on gold slot grids. Immunolabelling of the grids followed the protocol of Wilson et al. (2015). Briefly, the grids were blocked on a drop of 1% bovine serum albumin in PBS for 30 min, before being placed on the MLG antibody (Biosupplies) (1:500 dilution) for 12 h at 4°C. The grids were then washed three times in PBS and twice in the blocking buffer for 2 min at each step before being placed on the secondary 18 nm anti-mouse gold antibody (Jackson Immunolabs) (1:200 dilution). Binding specificity controls were performed with no primary antibody and only secondary antibody washes as described. Following immunolabelling, the sections were post-stained with 1% aqueous uranyl acetate for 10 min then lead stain for 2 min. The sections were imaged on a CM120 BioTwin transmission electron microscope (Thermo Fisher Scientific/FEI).

### Homology Modelling of the *BdCSLF6* A656T Mutant

An homology model of the *Bd*CSLF6 A656T variant was created to the BcsA catalytic subunit (PDB 4P00) (Morgan et al., 2013) using predicted structural alignments using HHpred (Zimmermann et al., 2018). The alignment was manually curated to allow gaps for regions of low homology which were excluded, such as the PilZ domain in the bacterial sequence, as well as the PCR and CSR of *Bd*CSLF6 whilst retaining the HHpred alignment for regions of predicted homology (Supplementary Figure 1). The homology model was generated using Modeller 9.14 (Webb and Sali, 2016), visualised and images rendered in Visual Molecular Dynamics 1.9.2 (Humphrey et al., 1996).

## Results

### Discovery and Screening for *BdCslF6* Mutants

In order to identify a reduced MLG variant of *B. distachyon*, a TILLING mutant population described by Dalmais et al. (2013) was screened for genetic lesions in *CslF6* causing amino acid changes predicted to lead to loss of function. A collection of twelve SNPs was identified by NGS which included five amino acid substitutions within the target region of *CslF6*, from amino acid T526 to F679 (Table 1), which contains key residues of the catalytic domain (Figure 1). Of those mutations, the most highly conserved residue positions amongst CESA/CSLs (Supplementary Figure 2) are W614, A656, and V667 which are either close to or within the crucial third Asp (xED) and QxxRW catalytic motifs, so plant lines with these variant residues were analysed in subsequent generations.

**TABLE 1:** TILLING lines identified by NGS to contain mutations resulting in amino acid substitutions in the target region of the *Bd*CSLF6 catalytic domain (Figure 1).

Line	Mutant	Description of mutation	No. of M3 individuals	Segregation ratio (Null:Het:Hom)
5431	A600T	In CSR domain	n.t	–
7573	G605E	In CSR domain	n.t	–
6495	W614*	Introduces stop before xED motif	24	1:0:0
5989	A656T	Behind the donor binding pocket	33	1:0:0
6076			16	1:0:0
7092			30	1:0:0
7175			32	11:1:1.5
7528	V667M	Variable residue in QxxRW motif	33	2.5:6:1

*One mutation at amino acid position A656 was detected in four independent lines, 5989, 6076, 7092, and 7175. The segregation ratio of the M3 individuals that germinated was determined by molecular genotyping by dCAPS (Supplementary Table 1). n.t., not tested; het, heterozygous; hom, homozygous.*

The mutation at W614 is particularly deleterious, introducing a stop codon resulting in a truncation before the essential xED and QxxRW catalytic motifs. The resulting polypeptide is predicted to be 67.5 kDa in size instead of the full length 105 kDa. The 24 progeny of M2 individuals from line 6,495 were genotyped in the subsequent M3 generation and no individuals were found to have retained the W614^∗^ mutation (Table 1). Mutants were also identified at position V667M, the first variable residue of the QxxRW motif (Table 1). Although a higher proportion of individuals at M3 retained the V667M mutation, none of the M4 progeny of two homozygous and two heterozygous individuals carried forward germinated so this mutation was also lost.

Retention of the A656T mutation at M3 was low, despite its presence in four independent M2 lines, 5,989, 6,076, 7,092, and 7,175, the number of null segregants was higher than expected, as for the other *cslf6* mutant lines (Table 1). Homozygous individuals were detected amongst the M3 seedlings that subsequently did not complete normal grain filling prior to senescence, producing severely shrunken grain, suggesting these mutations in *CslF6* impact negatively on seed set. In addition, severely stunted growth of individuals homozygous for A656T, and the absence of any viable seed at M4 also indicated that this mutation was detrimental to development. This was surprising given the relatively well tolerated *bgl* mutations in barley which showed normal germination rates, and only moderate reductions in mature height (Tonooka et al., 2009; Taketa et al., 2012) as similarly observed in the rice TDNA mutant (Vega-Sánchez et al., 2012). The low seed set at M3, absence of any viable seed at M4 and arrested growth observed in homozygous A656T mutants, as well as the lack of surviving homozygotes in other mutant lines, means that establishing a backcrossed line for future transgenic studies is not viable. However, further investigation of the physiological impacts of loss of MLG synthase capacity in A656T was conducted to provide insight into the apparent severity of this mutant and direct future research into storage polysaccharide regulation in this model species.

### Phenotypic Characterisation of the A656T Line

To further explore the impacts of loss of *CslF6* function on growth and development, A656T-7175 heterozygotes were investigated as they represent a less severe phenotype. A heterozygous individual from M3 was allowed to self-pollinate, and heterozygous progeny carried through in the subsequent M4 and M5 generations to generate material for analysis. To account for the variation that exists in the TILLING population from other background lesions, a Null-7175 sibling line was also established from an M3 sibling which was a null-segregant for A656T and carried through in the same way for comparison.

**FIGURE 2:**
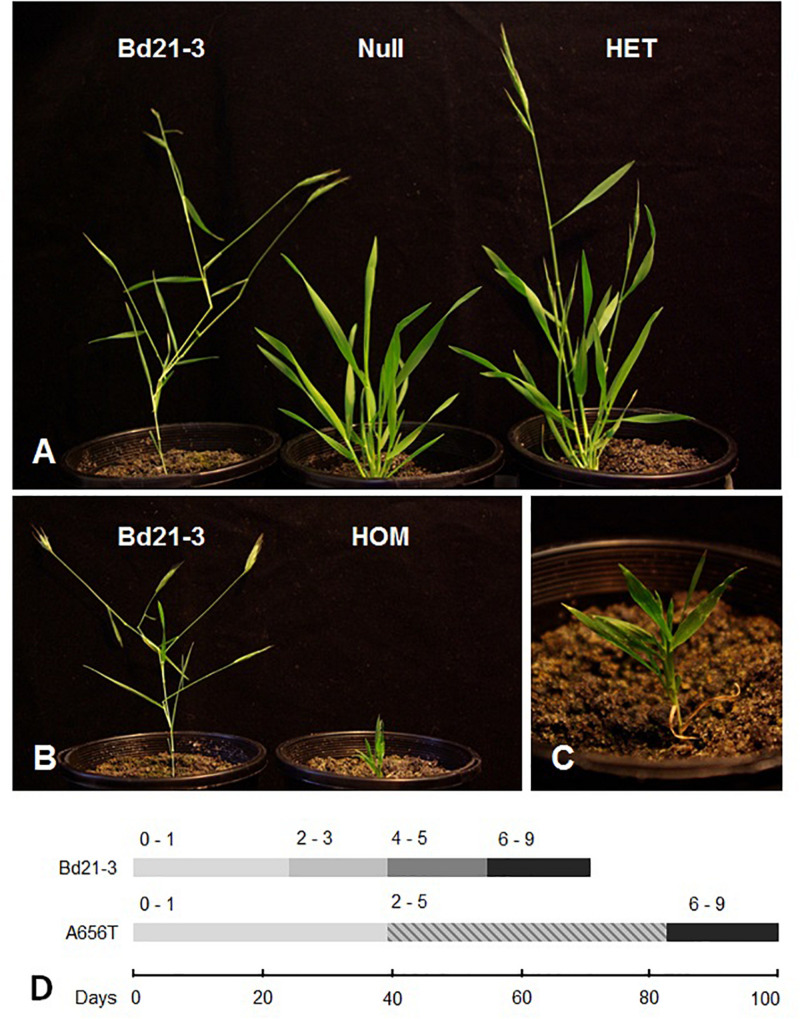
A656T 7175 mutants after 2 months of growth compared to wildtype Bd21-3. **(A)** A wildtype Bd21-3 individual (left) compared to a M6 null (centre) and heterozygous individual (right) which has entered the stem elongation phase. **(B)** Homozygous A656T mutant at 2 months old compared to a wildtype Bd21-3, both of which have reached their final heights, and **(C)** detail of compact arrangement of leaves yet to emerge from the sheath. **(D)** Timing of entry into key developmental stages on a modified BBCH scale (Hong et al., 2011); from germination and leaf formation (0–1); elongation of stem and tillering (2–3); heading and booting (4–5); to grain filling and senescence (6–9).

When the growth of segregating M5 and M6 generation A656T-7175 individuals was monitored for the entire life cycle and compared with Null-7175 and Bd21-3 plants mutant homozygotes displayed severely reduced growth. Monitoring of developmental progression (Figure 2D) found growth of homozygotes arrested at the early leaf formation stages (BBCH 0-1), in most cases failing to enter tillering and elongation phases (BBCH 2-3), or form grain (Figures 2B,C). Although still possessing background mutations, the stunted growth phenotype in A656T-7175 segregated with the *CslF6* genotype, with the heterozygous plants showing an intermediate growth rate, delayed entry into tillering and stem elongation (BBCH 2-3) and reduced final height compared to null segregant siblings, although were slightly taller on average than Bd21-3 plants at senescence (Figure 2A). Whilst these data are preliminary, that the null segregant siblings in A656T-7175 for consecutive generations displayed normal growth suggests broadly that MLG is important across the tillering and grain filling stages of development.

### Heterologous Expression of *BdCSLF6* Variants in *N. benthamiana*

**FIGURE 3:**
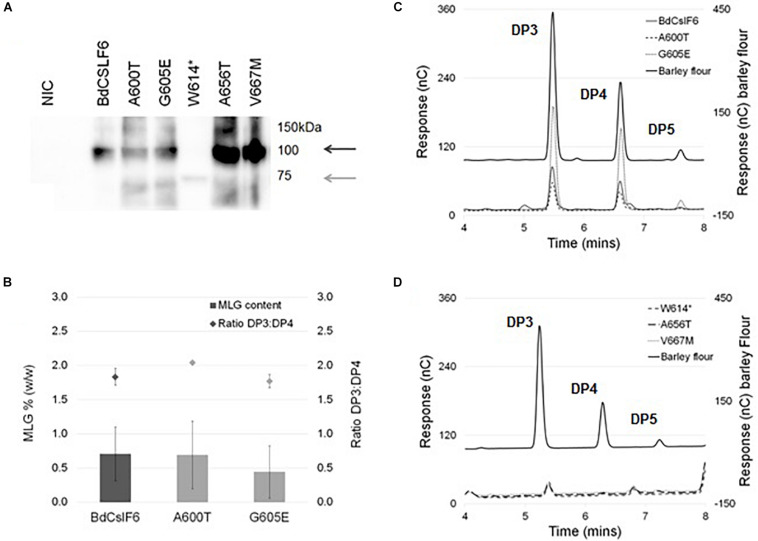
Heterologous expression of *Bd*CSLF6 variants present in TILLING lines in *N. benthamiana*, and analysis of the MLG content in resulting AIR preparations. **(A)** Representative immunoblot probed with the CSLF6 antibody of microsomal membranes prepared from transformed *N. benthamiana* leaves with either the native *Bd*CSLF6 or mutant sequence, harvested 2 DPI compared with a no infiltration control (NIC) (40 μg total protein). Dark grey arrow indicates expected size of the full length *Bd*CSLF6 protein at 105 kDa, and light grey arrow shows expected size of the W614* mutant which results in a truncated protein (approximately 67.5 kDa). **(B)** MLG content as a percentage of the dry weight of AIR preparations from *N. benthamiana* leaves (left *y*-axis), normalised to the abundance of wildtype BdCSLF6 protein (the same sample was run on each immunoblot), for variants which produced amounts detectable by HPAEC-PAD (*n* = 3, ± *SE*). Ratios between the amount of DP3 and DP4 are shown above each total percentage (± *SE*) (right *y*-axis). **(C)** Example of the HPAEC-PAD elution profile of lichenase digested AIR for variants found to produce MLG compared to a barley flour control (Megazyme); and **(D)** variants where no lichenase released products were detected. Characteristic oligosaccharide series with DP3, 4, and 5 indicated above peaks **(C,D)**.

To determine the impact of each mutation on MLG synthase activity of *BdCslF6*, each variant was expressed transiently in *N. benthamiana* leaves which do not produce any endogenous MLG, and have been used in previous *in vitro* studies to test CSLF6 function (Jobling, 2015; Wilson et al., 2015; Dimitroff et al., 2016). Leaves infiltrated with either of the two variants within the CSR, A600T, and G605E, were found to have an average of 0.69 and 0.44% (w/w) MLG in AIR preparations, respectively, not significantly different (ANOVA, α > 0.05) from the 0.70% produced by the wildtype *Bd*CSLF6 (*n* = 3) (Figure 3B). The ratio of DP3 to DP4 oligosaccharides released by treatment with lichenase, an enzyme that specifically cleaves the β-(1,4)-glucosidic linkage on the reducing end side of β-(1,3)-linked glucose residues within MLG chains, was also similar between the wildtype sequence, A600T and G605E variants at between 1.8 and 2.0 (Figure 3B). These ratios are comparable to those previously reported for *N. benthamiana* leaves expressing *Bd*CSLF6 (Jobling, 2015), and indicate the A600T and G605E variants have similar activity to the wildtype sequence.

The truncated W614^∗^protein expressed at a markedly lower level than the full length variants (Figure 3A), at an average of 9% relative to native *Bd*CSLF6, and was not found to produce detectable levels of MLG (*n* = 3) (Figure 3D), unsurprising given the extent of disruption to the active site. Similarly, the A656T and V667M mutations did not produce detectable lichenase-liberated oligosaccharides characteristic of MLG (Figure 3D), although in both cases the variant protein expressed at high levels, up to 3 or 4 times that observed in *Bd*CSLF6 native controls (Figure 3A). This strongly suggests that the proteins expressed with either the A656T or V667M mutations were not catalytically active.

### Homology Modelling Predicts A656T Effects Catalytic Function

**FIGURE 4:**
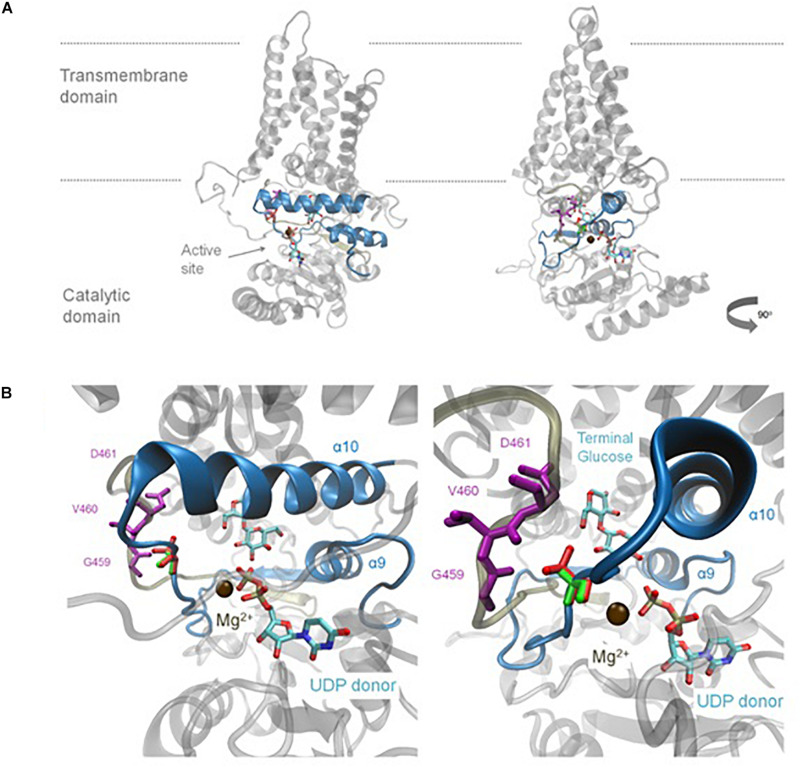
Homology model of the mutant *Bd*CSLF6 A656T protein showing the variant residue relative to the active site. Model shown in two rotations **(A)**, the relative position of the plasma membrane indicated with grey lines. Last two Glc residues of the nascent polymer, the acceptor, and recently transferred donor Glc are shown for illustrative purposes, which are joined by a β-(1,4)-linkage and shown relative to the UDP donor as captured in the structure described by Morgan et al. (2013). **(A)** The UDP donor is still bound in the active site, coordinated by a Mg^2+^ ion shown in brown. The region which corresponds to the NGS screen for mutations in the TILLING population is indicated in blue excluding the CSR. **(B)** Enlarged view of the region of the donor binding pocket in relation to the UDP and nascent chain where the A656T mutation models. The native Ala at position 656 is shown in green, compared to the Thr substitution in red **(B)**. The mutation is present in a flexible region between α helices 9 and 10 and is within 5 Å of several residues in the loop behind, G459, V460, and D461, shown in purple **(B)**.

Homology modelling to the solved BcsA structure (Morgan et al., 2013), was used to provide insight into the possible impact of the A656T mutation within the predicted structure of *Bd*CSLF6. Whilst the plant-specific CSR residues in the TILLING could not be included, lacking homology to BcsA, the A656T mutation is predicted to lie close to the UDP-Glc donor (Figure 4). Residue A656 lies on a flexible loop between alpha helix 9 (α9), which includes the predicted catalytic residue in the xED motif, and α10 where the QxxRW coordinates the acceptor Glc on the nascent chain (Figure 4B). The change at this residue from Ala to Thr introduces a polar sidechain which the homology model predicts would face back to the loop that packs in behind (Figure 4B). Three residues, G459, V460, and D461 (Figures 4B,C) are within 5 Å of residue 656, and therefore could interact. Based on the model, it is likely that a change in residue 656 would impact the interaction of these loops and could change their ability to coordinate the UDP-Glc substrate and magnesium cation co-factor, which may explain why *Bd*CSLF6 A656T is inactive.

### The A656T-7175 Line Shows Reduced MLG in the Grain

To investigate the impact of loss of function of *CslF6* on growth *in vivo*, the MLG content of grain was compared between lines. An average reduction in MLG of 21% (w/w) was observed in pooled M6 grain from the heterozygous A656T-7175, concurrent with a more than 2.5-fold increase in total starch (% w/w) compared to the wildtype (Table 2). The Null-7175 grains showed an 8% higher MLG content and 10% higher starch content than wildtype, however the MLG:starch ratio was very similar between lines at 4.6 and 4.5, respectively. This indicates they are proportionately similar despite a slight difference in grain weight, which was not found to be significant (ANOVA α > 0.05, *n* = 5) (Table 2). In contrast, A656T-7175 showed a major reduction in the ratio of MLG:starch to 1.5 due to the proportionate shift in both polysaccharides (Table 2). The content of MLG, as well as the ratios of MLG:starch in the Bd21-3 are consistent with those previously reported for this line (Trafford et al., 2013), indicating the change in A656T-7175 is likely due to the mutation in *CslF6*.

**TABLE 2:** The MLG and starch content of M6 grain from A656T 7175 heterozygotes compared with Null 7175 and wildtype Bd21-3.

Line	Ave grain weight (mg ± SE)	MLG (% w/w)	Starch (% w/w)	Ratio MLG:Starch
Bd21-3	4.29 ± 0.19	17.30	3.73	4.6
Null 7175	5.00 ± 0.21	18.74	4.14	4.5
A656T 7175	4.49 ± 0.23	13.76	9.33	1.5

*Average grain weights (mg) are shown, and were not found to differ significantly between lines (ANOVA α > 0.05, n = 5).*

Quantitative RT-PCR was used to examine if the transcript abundance of other *CslF* and *H* isoforms changes in response to A656T. To capture the beginning of increased MLG deposition in the endosperm cell walls following cellularisation (Guillon et al., 2011), and the reported peak of *Bd*CSLF6 transcript in grain (Trafford et al., 2013), transcripts of the *CslF* and *H* isoforms were measured at 8–10 DAP in A656T heterozygous grain, Null-7175, and wildtype Bd21-3 lines. As previously reported, *CslF6* and *F8* were the most highly expressed isoforms in developing grain (Trafford et al., 2013), alongside *CslF4* and *F10-1* which were similarly abundant in Bd21-3 (Supplementary Figure 3). For most *Csl* isoforms, including *CslF4*, *F9*, *F10-1*, *F10-2*, and *H1*, normalised transcript abundance was lower in A656T-7175 than both the Null-7175 and wildtype (Supplementary Figure 3), however, this variance between lines was not found to be statistically significant for any genes measured (two factor ANOVA, α > 0.05). These data do not support the notion that there is a change in the transcription of any *CslF* or *H* isoforms driven by differences between the lines either from background mutations or due to partial loss of CSLF6 enzymatic capacity due to the presence of the A656T mutation in the 7175 line.

### A656T-7175 Grains Show Altered Endosperm Cell Architecture

**FIGURE 5:**
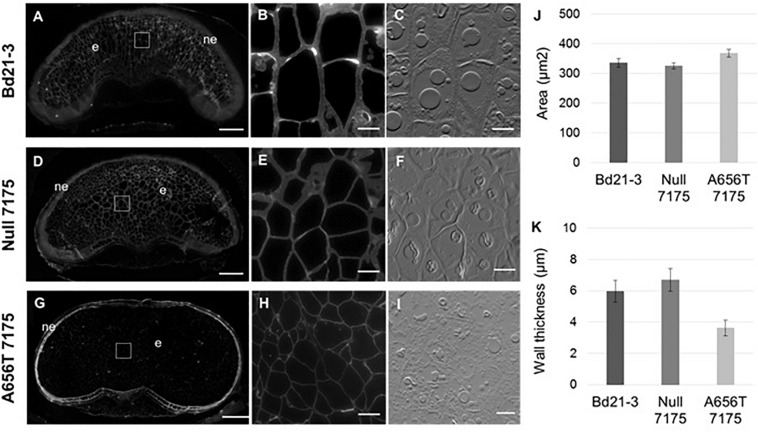
Comparison of MLG content and deposition in mature grains between wild type Bd21-3, Null 7175, and A656T 7175 lines. Fluorescent antibody labelling of MLG on resin sections with identical light intensity and exposure times **(A–I)**. Transverse sections of whole, mature grain at 18–20 DAP from the Bd21-3 **(A)**, Null 7175 **(D)**, and A656T 7175 **(G)** lines, scale bars shown at 200 μm, nucellar epidermis (ne), and endosperm (e) are indicated. Labelling is evident in the walls of endosperm cells **(B,E,H)** with adjacent interference contrast brightfield images shown for cellular detail **(C,F,I)**, scale bars show 20 μm. **(J)** Average cell areas (μm^2^) of endosperm cells for Bd21-3 (*n* = 837), Null 7175 (*n* = 1,261) and A656T 7175 (*n* = 1,103) (± SE). **(K)** Average cell wall thickness of endosperm cells from Bd21-3 (*n* = 21), Null 7175 (*n* = 40) and A656T 7175 (*n* = 53).

Fluorescent antibody labelling of MLG was performed on grains from all lines at 18–20 DAP when grain filling is complete but before plants enter senescence, to capture the maximum MLG levels in mature grain. Labelling showed MLG present in both the walls of the endosperm, as well as the nucellar epidermal layer in Bd21-3 wildtype (Figure 5A) and Null-7175 (Figure 5D). Although the labelling within the nucellar epidermal layer of A656T-7175 grain (Figure 5G) appeared more intense compared to that of wildtype and Null-7175 (Figures 5A,D), this is an artefact of the lower intensity observed overall due to reduced labelling in endosperm walls (Figure 5G). This suggests the impact of reduced MLG synthase capacity in mutants was greatest in the endosperm than any other tissue in the grain.

At higher magnifications the walls of endosperm cells labelled with MLG appeared to be thicker in Bd21-3 (Figure 5B) and Null-7175 (Figure 5E) than in the equivalent cells of A656T-7175 grains. Across the endosperm the size of cells varied, although tended to appear larger in A656T-7175 grain than for the other lines (Figure 5H). To investigate this observation, both the average cell area and wall thickness of endosperm cells were measured and compared between lines. Whilst a slight increase in the average endosperm cell area in A656T-7175 was measured compared with Bd21-3 and Null-7175 (Figure 5J), there was a marked reduction in wall thickness in mutants, which were approximately 30% thinner on average (Figure 5K).

**FIGURE 6:**
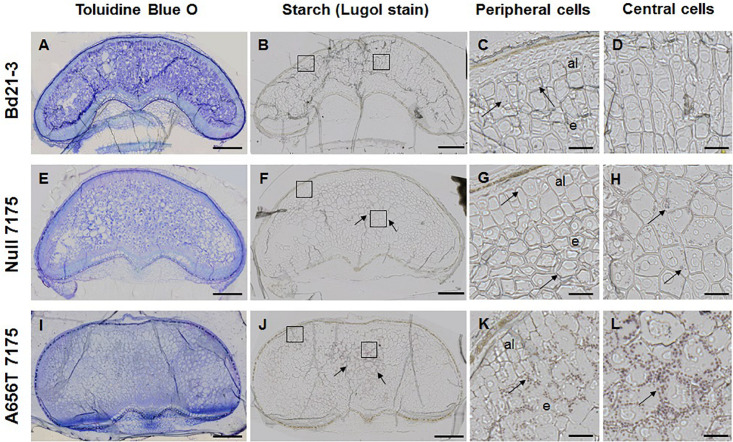
Starch granules in the endosperm of wildtype Bd21-3 grain compared with Null 7175, and A656T 7175, in different sections from the same grains shown for MLG (Figure 5). **(A,E,I)** TBO staining showing general architecture of grains in transverse sections, collected at maturity (18–20 DAP). Lugol staining for starch in whole grains of Bd21-3 **(B)**, Null 7175 **(F)**, and A656T 7175 **(J)**. Scale for images of whole grains shown as 200 μm. Detail showing starch grains stained with Lugol within cells, indicated with black arrows, at the periphery of the grain including aleurone (al) **(C,G,K)**, and in the central endosperm (e) for Bd21-3 **(D)**, Null 7175 **(H)**, and A656T 7175 **(L)** lines. A 20 μm scale is shown for the higher magnifications **(C,D,G,H,K,L)**.

Given the observed increase in the starch content in whole grains of A656T-7175 (Table 2), mature grain was also stained with Lugol reagent to observe starch granule formation (Figure 6). In wildtype grain starch staining was low overall (Figure 6B), with small granules observed in the aleurone and in some cells at the periphery of the endosperm, but rarely in the central cells (compare Figures 6C,D). More staining was observed in Null-7175 grain (Figure 6F), which were more variable compared with Bd21-3 wildtype (Figure 6B), with small starch granules observed more frequently in the central cells of the endosperm as well as in the aleurone (Figures 6G,H). In A656T-7175 grain more starch staining was observed (Figure 6J) than in either wildtype or Null-7175 grains, and this was concentrated in the central cells of the endosperm with larger and more numerous granules than the aleurone (Figures 6K,L). Although no difference was observed between lines in the amount of starch observed in aleurone layers, there was more starch present in larger granules of A656T-7175 central endosperm cells than Null-7175 or wildtype grains.

The possibility that A656T-7175 individuals may also display other differences in their endosperm cell walls in response to decreased deposition of MLG was also explored through immunolabelling for cellulose (S4B), unbranched xylans by detection of the non-reducing ends of xylan backbone chains (LM10) and branched xylans (LM11) (Supplementary Figure 4). In rice *cslf6* mutants coleoptiles were found to have slight increases in unbranched xylans in response to reductions in MLG (Vega-Sánchez et al., 2012). Strong labelling for cellulose was observed in *B. distachyon* grain within endosperm, nucellar epidermis, and pericarp cell walls, and was at similar intensities in all lines (Supplementary Figures 4J,P,V). Some labelling for unbranched xylans was observed in pericarp, however no xylan labelling was observed in the endosperm walls of any line (Supplementary Figures 4K,L,Q,R,W,X). Together these results indicate that no large observable differences in polysaccharide distribution occur in the walls of A656T-7175 endosperm cells in response to reduced MLG deposition.

### Homozygous A656T Mutants Show Reduced MLG in First Stem Internodes

**FIGURE 7:**
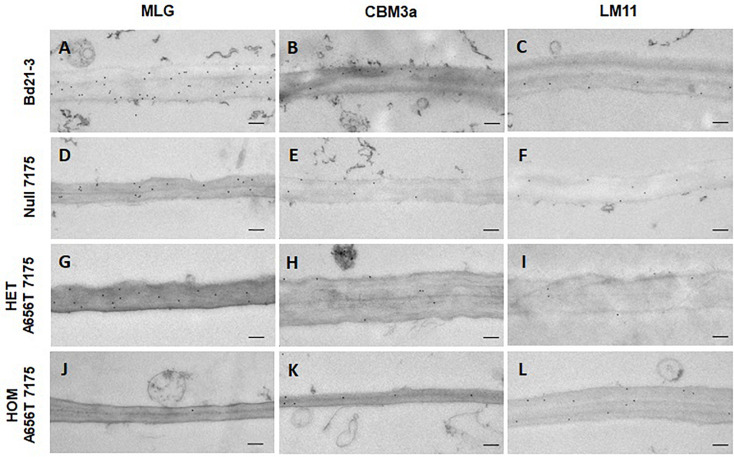
TEM and immunogold labelling of first internode of stems from all lines upon entering elongation phase at 3 weeks post germination. Cell walls of central pith cells labelled with antibodies for MLG **(A,D,G,J)**, cellulose (CBM3a) **(B,E,H,K),** and branched xylans (LM11) **(C,F,I,L)**. The wildtype Bd21-3, Null 7175, heterozygous A656T-7175, and homozygous mutant are compared as shown on the left. Scale bars shown as 200 nm.

To investigate the impact of the A656T mutation on vegetative tissues, given the observed impacts on growth in homozygotes, stems were sampled, and the deposition of MLG in walls analysed by TEM (Figure 7). The MLG content in mature stems of *B. distachyon* is expected to be moderately high in wildtype relative to rice (Vega-Sanchez et al., 2013), and may therefore be important during the stem elongation phase where growth arrested in homozygous mutants (Figure 2D). The first internode was sampled as this tissue showed relatively high expression of *CslF6* in the transcript map (Sibout et al., 2017), at 3 weeks after germination to capture the expected entry into tillering and stem elongation phases (BBCH 2-3) (Hong et al., 2011).

All lines showed MLG present in the walls of the first internodes, however a large difference in labelling was observed between wildtype and homozygous mutants in the central pith cells (Figures 7A,J). Walls in the central pith of wildtype Bd21-3 labelled with an average of 24 gold particles per μm^2^ of wall, whereas homozygotes had greatly reduced MLG with an average of 1.4 gold particles per μm^2^ (Figures 7A,J). This suggests a significant reduction in the MLG content of pith cells in individuals homozygous for A656T may contribute to the inability of these plants to support the normal development of stems during growth. In addition, the walls of homozygous internodes appeared thinner, and displayed more abnormally curved shapes and folds compared with wildtype. The deposition of MLG observed in Null 7175 and heterozygous mutants was similar (Figures 7D,G), and consistent with the observation that these lines were able progress through the normal stages of development, although heterozygous individuals were on average delayed in entry into stem elongation (Figure 2A). Labelling of cellulose (CBM3a) and branched xylans (LM11) in the first internodes showed similar deposition across all lines, providing no evidence that the composition of these polysaccharides was altered (Figure 7).

## Discussion

MLG is a soluble dietary fibre that has been linked with a number of health benefits including lowering the risk of colorectal cancer, cardiovascular disease, and reducing cholesterol reabsorption (Collins et al., 2010; Othman et al., 2011; Daou and Zhang, 2012; Gunness et al., 2016; Wang et al., 2016; Johnson et al., 2018). The *Himalaya 292* starch synthase mutant of barley with lowered production of total starch has an increased content of both resistant starch and soluble non-starch polysaccharides, including β-glucans (MLG) (Bird et al., 2004a, b). The MLG and other soluble non-starch polysaccharides are both considered components contributing to the increased soluble dietary fibre in the grain which have been shown to improve indices of general bowel health (Bird et al., 2008; McOrist et al., 2011) and result in lower plasma insulin levels (King et al., 2008) in humans.

*B. distachyon* is of particular interest in understanding the biosynthesis of MLG and the implications of this for manipulation of storage polysaccharides to improve the health and nutritional value of related grains, given its distinctive endosperm composition. *B. distachyon* has a notably high content of MLG at 45% (w/w) of grain weight and low, 6% (w/w) content of starch (Guillon et al., 2011), compared to other domesticated cereals where starch is the predominant carbohydrate. In contrast to similar *cslf6* mutants in rice (Vega-Sánchez et al., 2012) and barley (Tonooka et al., 2009; Taketa et al., 2012), loss of CSLF6 synthase capacity in *B. distachyon* mutant A656T-7175 led to severely arrested growth and delays in tiller development in particular (Figure 2). The MLG content in endosperm was reduced by 21% (w/w) in A656T-7175 compared with wildtype, and concurrent with a 2.5-fold increase in starch (% w/w) resulting in a much lower MLG:starch ratio in *cslf6* mutants (Table 2). This suggests that understanding the regulatory mechanisms governing carbon partitioning between intra- and extracellular polysaccharide storage and metabolism in *B. distachyon* grain could provide an additional avenue to manipulate the overall nutritional value of grains for human consumption.

With the advantage of a well-defined GT2 catalytic domain, the targeted approach deployed in the NGS screen of a 463 bp region of *BdCslF6*, which includes the xED and QxxRW motifs successfully identified five amino acid substitutions (Figure 1). The finding that three variants W614, A656, and V667, each at highly conserved residues amongst grass *CslF6* isoforms (Supplementary Figure 2), caused a loss of catalytic function when expressed *in vitro*, producing no detectable MLG in AIR preparations of *N. benthamiana* leaves, validated this approach to identification of mutants (Figure 3). Interestingly, two variants within the CSR, A600T, and G605E, were able to produce MLG with a ratio between DP3 and 4 similar to both the wildtype control and to that previously reported (Jobling, 2015), indicating mutations were tolerated in this region (Figure 3B). Whilst the A600 position is more conserved amongst grasses than G605E (Supplementary Figure 2), both lie within the CSR region which has unknown function, although it has been suggested to be involved in either oligomerisation or protein–protein interactions based on current models for CESA rosette formation (Delmer, 1999; Sethaphong et al., 2013; Olek et al., 2014), and contains residues under selection in *HvCslF6* (Schwerdt et al., 2015; Supplementary Figure 2). In contrast, the disruption of key catalytic residues in the QxxRW motif in the W614^∗^ and V667M variants were predictably detrimental and not able to produce MLG *in vitro* (Figure 3D). Although A656 is a residue which is conserved amongst CESA/CSLs, the likely reason for the loss of function observed *in vitro* in the A656T mutation was proposed from homology modelling to the BcsA structure which placed it within the UDP-Glc binding pocket (Figure 4). Within 5 Å from the residues G459-D461 in the flexible structure at the back of the binding pocket (Figure 4B), modelling suggests that the introduction of Thr with a polar side group projecting backward causes disruption of the pocket structure.

The lower than expected segregation ratios of *cslf6* mutant genotypes observed within developing grains in early mutant generations across several independent lines suggested that loss of *CslF6* catalytic function may be more deleterious to normal development in *B. distachyon* than for either barley or rice. This greatly reduces the usefulness of a stable *cslf6* mutant in *B. distachyon* for transgenic studies of MLG synthesis, however inducible or tissue-specific mutants may be an alternative option. Although a reduction in seed set of 50% was also reported in rice *cslf6* TDNA mutants (Vega-Sánchez et al., 2012), and a 30% reduction in grain yield for *bgl* barley (Taketa et al., 2012), similar mutations in *B. distachyon* were found to be particularly detrimental. In rice TDNA *cslf6* mutants anthers and filaments were found to be deformed, leading the authors to suggest partial male sterility may influence reduced seed set (Vega-Sánchez et al., 2012). If similar developmental abnormalities result in *B. distachyon* this could explain the low rates of homozygosity as well as reduced capacity to form grain in A656T, although the magnitude of this effect is greater than for rice. Even when homozygous A656T-7175 individuals were able to germinate successfully, at a rate of approximately 10%, mature plants were severely stunted (Figures 2B,C), in most cases unable to enter stem elongation and tillering phases of growth, or produce grain. Where grain was produced, they appeared severely shrunken and did not result in any successful germinations beyond the M4 generation. *CslF6* in wildtype *B. distachyon* is most highly transcribed in roots, shoots, first node and internode, last node and internode, young leaves, young spikelets as well as developing grain (Sibout et al., 2017), broadly consistent with the growth patterns observed in seedlings and grains in this study. During validation of a TDNA population of *B. distachyon* Hsia et al. (2017) reported an approximately 50% reduction in MLG in whole seedlings of *cslf6* mutants, which by 3 weeks of growth displayed a reduced average height of 20% of wildtype. This is consistent with both the severely impaired growth of A656T-7175 homozygotes, and transcriptomic evidence for the role of *CslF6* in leaf and stem (Sibout et al., 2017) at the early stages of growth. After 3 weeks of growth wildtype plants should have entered the tillering and stem elongation stages (Hong et al., 2011), however the impact of the loss of *CslF6* in later stages was not reported for these TDNA mutants as it was beyond the scope of the study (Hsia et al., 2017). In wildtype rice the abundance of MLG in stems is higher than in *B. distachyon* (Vega-Sanchez et al., 2013), which may indicate that reductions in MLG synthase capacity could be expected to have more severe impacts on development of tillers that the moderate 33% reduction in height for rice *cslf6* mutants (Vega-Sánchez et al., 2012). Contrary to this expectation, the walls of central pith cells in homozygous A656T-7175 mutants showed greatly reduced MLG deposition at 1.4 gold particles per μm^2^ of wall compared with 24 gold particles per μm^2^ of wall in wildtype (Figures 7A,J) reflecting the observed arrested growth before stem elongation (Figure 2D). In the less severe heterozygous mutants however, the deposition of MLG was similar to Null 7175 and wildtype indicating some loss of CSLF6 synthase capacity was tolerated allowing for stem elongation (Figures 7D,G). The severity of the impacts on homozygotes suggests that MLG may play distinct, additional roles within *B. distachyon* tissues compared to rice and loss of *CslF6* is particularly detrimental to stem development preventing progression through normal development.

The comparative severity of loss of function mutants may be a consequence of larger proportionate reductions in MLG in grains where wildtype contains much higher MLG per grain weight than other cereals. In barley *bgl* mutants for example, the content of MLG in grain was reduced from 3.2% (w/w) to undetectable levels (Tonooka et al., 2009), however, this is more than five times less MLG in wildtype grain compared with Bd21-3 which had a content of 17.3% (w/w) (Table 2), and can be up to 45% (w/w) of grain in other *Brachypodium* lines (Guillon et al., 2011; Trafford et al., 2013). Grain produced by the A656T-7175 heterozygotes had an average 21% (w/w) reduction compared to wildtype, and 26% (w/w) less than Null-7175 (Table 2). The reason for this difference in MLG contents in wildtype *B. distachyon* compared with barley remains unclear, as the expression of *CslF6* was not found to differ greatly on average between species across grain development (Trafford et al., 2013), so the high total MLG content of grains does not result from differences in transcriptional regulation of *CslF6* alone. Analysis of the transcript levels of CslF and H genes at 8–10 DAP, the reported peak of CslF6 expression in *B. distachyon* grain development (Trafford et al., 2013), did not show any significant differences between the lines for any gene, suggesting no obvious compensation occurs at the transcriptional level for the partial loss of MLG synthase capacity (Supplementary Figure 3). Together this these data are consistent with a non-redundant role for BdCslF6 in MLG synthesis in *B. distachyon* grain, which is aligns with barley *bgl* mutants where no increase in *CslF6* transcription is observed (Taketa et al., 2012), and in rice where no CslF or CslH isoform showed higher expression in TDNA mutants (Vega-Sánchez et al., 2012). The lack of evidence for transcriptional changes in the *Csls* combined with the observation of increased starch content in MLG deficient A656T 7175 grain (Table 2) suggests that mechanisms controlling carbon partitioning between these two polysaccharides may have a great impact on their abundance.

The much lower ratio of MLG:starch at 1.5 in A656T-7175 compared to wildtype and Null-7175 with ratios of 4.6 and 4.5, respectively (Table 2), suggests a redistribution of carbon resources toward starch in response to lowered MLG synthase capacity, supporting a proposed role for MLG as an alternative source of accessible sugars during germination (Fincher, 2009a; Guillon et al., 2011; Trafford et al., 2013). However, the arrested growth of A656T mutants could indicate that MLG and starch are not entirely physiologically equivalent glucose reserves during *B. distachyon* germination. A compensatory increase in MLG is also observed in *lys5*, a starch-deficient mutant of barley, which shows up to a 30% reduction in total starch compared with wildtype depending on the parental line (Christensen and Scheller, 2012), likely due to an inability to transport the ADP-Glc donor into the plastid for synthesis (Patron et al., 2004). This points to a diversion of Glc from ADP-Glc required for starch synthesis toward UDP-Glc for MLG synthesis. However, the observation that *lys5* mutants display low transcript abundance for *CslF6* at late stages of endosperm development compared to wildtype indicates this process likely involves some transcriptional regulation in response to lower MLG levels in the wall in barley (Christensen and Scheller, 2012). The interrelated regulation of MLG and starch synthesis has also been shown conversely in barley lines overexpressing the β-D-(1,3;1,4)-glucanase isoenzyme EII (*HvGlb2*), an enzyme that hydrolyses MLG, where the expected reduction in grain MLG also resulted in a parallel increase in starch (Han et al., 2017). It is therefore likely that a similar mechanism occurs in *B. distachyon* where the reduced MLG synthase capacity in mutants possessing the A656T mutation triggers a compensatory increase in starch synthesis to divert the Glc into this alternate storage polysaccharide. Furthermore, quantitative trait loci (QTL) associated with MLG content of tetraploid wheat include regions encoding starch synthase II and the hydrolytic enzymes isoamylase (GH13) and β-amylase (GH14) (Marcotuli et al., 2016), supporting that regulation of both carbohydrates is connected. Indeed, there is evidence that the sucrose synthase regulatory network controlling supply of both ADP-Glc and UDP-Glc links synthesis of other cell wall polysaccharides such as cellulose with starch production (Stein and Granot, 2019). The alterations to the MLG:starch ratios observed in A656T-7175 should direct future work to further investigate a likely cross-regulation of the two synthesis pathways. It would be of particular interest to determine whether the regulatory mechanisms are similar to or distinct from barley and wheat, although these regulatory networks are not yet fully understood. Given current evidence, it is likely that cross-regulation of MLG and starch involves multiple levels of control, from gene expression to enzyme activity, within pathways for substrate synthesis and transport, as well as both synthesis and turnover of the polysaccharides.

Fluorescent antibody labelling of MLG in grains 18–20 DAP reveals a role for *CslF6* in the development of endosperm cells, confirming alterations to content and revealing differences in cell morphology in A656T-7175 grains (Figure 5). Reduced labelling across the endosperm of A656T-7175 (Figures 5G,H) likely accounts for the overall lower MLG content of whole grains (Table 2) compared to wildtype. The reduction in MLG resulted in altered morphology of the endosperm in A656T-7175 showing a slightly larger cell area (Figure 5J) and approximately 30% reduction in average wall thickness (Figure 5K). This altered morphology is particularly interesting given the concurrent increase in starch deposition in the endosperm of these mutants (Figures 6J–L), which has previously been suggested to influence cell enlargement in later stages of grain development in *B. distachyon* in a comparative study with barley (Trafford et al., 2013). Whilst the endosperm walls of *B. distachyon* are characteristically thicker than other cereals (Guillon et al., 2011; Tanackovic et al., 2014), Trafford et al. (2013) note this occurs in the later stages after cellularisation, when both MLG and starch deposition increase, but a slower rate of cell enlargement results in smaller cells at peak fresh weight than in barley. Although the area of the cytoplasm, not including starch granules, is similar between both species in endosperm cells, and does not change significantly during cell enlargement, the area occupied by starch granules is greater in barley, accumulating to fourfold the total amount found in *B. distachyon* mature grain (w/w) (Trafford et al., 2013). It was therefore proposed that starch accumulation may drive cell enlargement in barley resulting in thinner walls and larger cell areas, but limits expansion in *B. distachyon* where starch production is lower, and cells do not need to accommodate its accumulation to the same extent. It remains unclear whether the thickened walls of *B. distachyon* endosperm contain more MLG per cell compared to barley (Trafford et al., 2013), given only a slight increase in the proportion of MLG from 80% (w/w) of endosperm wall in *B. distachyon* compared with 75% (w/w) in barley (Guillon et al., 2011).

Although the increase in endosperm cell area in A656T-7175 mutants was slight (Figure 5J), it supports the proposition that cell enlargement is facilitated by increased starch accumulation resulting from reduced MLG deposition. The larger starch granule size observed in endosperm cells of A656T-7175 (Figures 6K,L) compared to wildtype (Figures 6C,D) and Null-7175 grain (Figures 6G,H) was an interesting finding. *B. distachyon* usually generates much smaller granules than other cereals, up to twofold or threefold smaller than barley and wheat (Opanowicz et al., 2011; Tanackovic et al., 2014). It has been suggested that smaller granule size may simply be a consequence of low starch deposition resulting from low transcriptional activity of starch synthase genes (Trafford et al., 2013; Tanackovic et al., 2014), and this would be supported by the observation that A656T-7175 produced more starch, in larger granules (Figures 6K,L). There is some evidence that the low transcriptional activity of starch synthases only occurs during early grain development, and lack of activation by post-translational modification also contributes to low starch in *B. distachyon* compared with wheat (Chen et al., 2014). However, reduced starch content may also be linked to altered starch composition in *B. distachyon*, which has slightly reduced amylose content compared to barley, although amylopectin branch patterning is very similar (Tanackovic et al., 2014). In the barley starch mutant, *sex6*, for example disruption of the starch synthase IIa (*SSIIa*) gene resulted in shrunken endosperm due to reduced starch production, but also altered composition with increased amylose levels compared to wildtype (Morell et al., 2003). Although *B. distachyon* has the full complement of genes required for starch production (Trafford et al., 2013; Tanackovic et al., 2014), the physiological purpose for the low production and slight compositional difference compared to high starch cereals is unknown. Further characterisation of the crystallinity and composition of starch produced in A656T-7175 would be required to ascertain if this genuinely represents a grain morphology that has shifted to be more like that of domesticated cereals.

Whilst establishing a stable *cslf6* mutant in *B. distachyon* for use in transgenic studies may not be a viable approach to gaining a further understanding MLG synthesis, the observed alterations to endosperm morphology and redirection of carbon resources toward starch in grain with reduced MLG levels have significant implications for future studies in this area. These implications are twofold; firstly, the changes we describe in morphology of grains with reduced MLG support the notion that cell enlargement during grain filling is reliant on the deposition of starch in *B. distachyon*, and is a key determinant for the differences in morphology and composition compared with crops such as barley and wheat. A detailed description of the regulation of starch deposition should be pursued to further evaluate *B. distachyon* as a model for temperate grasses. Secondly, the possibility of cross-regulation of MLG and starch deposition in *B. distachyon* endosperm has important consequences for manipulating the nutritional content of other cereal grains. Establishing the nature of any cross-regulatory network of these two polysaccharides could indeed direct breeding or biotechnological approaches to altering the ratio of starch to soluble dietary fibre content of commercially important cereal grains.

## Conclusion

Characterisation of a *cslf6* mutant, A656T, which showed loss of function *in vitro*, revealed severely arrested growth and delayed development suggesting a critical and non-redundant role for *CslF6* in *B. distachyon*. Reduced MLG in grain was concurrent with increases in starch deposition, rather than major changes in cell wall polysaccharide distribution, as well as changes to endosperm architecture including thinner cell walls and larger starch grains representing a morphology more similar to barley and wheat grain. This suggests the partitioning of carbon resources in *B. distachyon* relies on a likely cross-regulation of MLG and starch synthesis pathways, and supports previous comparative studies that posit that the interplay between the deposition of these polysaccharides in grain is a key driver of interspecies variation. Investigation of the regulation of MLG and starch should therefore be a focus of future studies within *B. distachyon* and may provide insight into how the regulation of these pathways contributes to grain carbohydrate content, an important consideration for the nutritional value and composition of cereals.

## Data Availability Statement

The raw data supporting the conclusions of this article will be made available by the authors, without undue reservation.

## Author Contributions

MD and MB conceived the project. MD supervised the work. AM performed the microscopy and image analysis. RC performed the sequencing and identification of mutants in TILLING screen. MB performed all the other data collection and analysis, including writing of the manuscript with input from other authors. All authors contributed to the article and approved the submitted version.

## Conflict of Interest

The authors declare that the research was conducted in the absence of any commercial or financial relationships that could be construed as a potential conflict of interest.

## Footnotes

1 www.CAZy.org

2 http://helix.wustl.edu/dcaps/dcaps.html
