# Supplementary material for: Characterisation of Cellulose Synthase Like F6 (CslF6) Mutants Shows Altered Carbon Metabolism in β-D-(1,3;1,4)-Glucan Deficient Grain in Brachypodium distachyon
Source: Front Plant Sci. 2021 Jan 11;11:602850. doi: 10.3389/fpls.2020.602850 (PMC7829222; doi:10.3389/fpls.2020.602850)
Supplement: Supplementary file 1 [file Data_Sheet_1.PDF]

Supplemental Information

Supplemental Table 1. Primers used for dCAPS marker genotyping of *B. distachyon* TILLING lines, including the enzymes specific for each derived restriction site created when primer anneals adjacent to each mutation. Expected digestion products were diagnostic for the presence of wildtype, mutant or both alleles compared to the Bd21-3 wildtype. The primers listed for screening of line CSLF6 7175 were also used for lines CSLF6 5989, 6076 and 7092 which contained the same SNP.

**Line**	**Mutant**	**SNP**	**dCAPS markers**			**Expected digestion products (bp)**		
			**Forward primer**	**Reverse primer**	**Restriction enzyme**	**Undigested**	**Wildtype**	**Mutant**
CSLF6 6495	W614*	G →A (1842)	CGGCATTCGAGCAGAAGACCGGGTG	GAAGAAGATCTCGAGGGAGC	HphI	221	143, 78	143, 49, 29
CSLF6 7175	A656T	G →A (1966)	GGCTGAGGCCGTCAAGG	AGCCGCTCCGTGAGGTTGATCGAGC	AluI	201	140, 61	116, 61, 24
CSLF6 7528	V667M	G →A (1999)	GGCTGAGGCCGTCAAGG	AGGGAGCCGGTGGACCAGCGGACCA	BccI	234	113, 79, 42	113, 47, 42, 32

Supplemental Table 2. Cloning primers used to generate TILLING variants for heterologous expression in *N. benthamiana*. Wildtype sequence was amplified from cDNA from Bd21-1 seedlings under the CaMV 35S promoter in a modified pGreen II vector (Wilson et al., 2015). Variants were generated from the wildtype *Bd*CslF6 construct in two fragments by inclusion of each SNP in the primer sequences, shown in lower case, and ligated with Gibson Assembly reagent (NEB).

**Variant**	**Forward primer**	**Reverse primer**
Wildtype *BdCslF6*	TCGAGGAATTCGGTACCATGGCGCCAGCGGTGG	GGACTCTAGAGGATCCTCACGGCCAGAGGTAG
BdCslF6-A600T 5' fragment	Wildtype *BdCslF6* Forward	CCTTGACGGtCTCAGCCAGAGTC
BdCslF6-A600T 3' fragment	GGCTGAGaCCGTCAAGGTG	Wildtype *BdCslF6* Reverse
BdCslF6-G605E 5' fragment	Wildtype *BdCslF6* Forward	GAATGCCGATtCGGTCACCTTGAC
BdCslF6-G605E 3' fragment	GGTGACCGaATCGGCATTCGAG	Wildtype *BdCslF6* Reverse
BdCslF6-W614* 5' fragment	Wildtype *BdCslF6* Forward	CGCTGCCtCATCCGGTCTTCTG
BdCslF6-W614* 3' fragment	GACCGGATGaGGCAGCGAGCTC	Wildtype *BdCslF6* Reverse
BdCslF6-A656T 5' fragment	Wildtype *BdCslF6* Forward	GGTTGATCGGGGtGGTGCCGATG
BdCslF6-A656T 3' fragment	GCACCaCCCCGATCAACCTCACGG	Wildtype *BdCslF6* Reverse
BdCslF6-V667M 5' fragment	Wildtype *BdCslF6* Forward	GAGCAtCTGGAAGAGCCGCTCCGTG
BdCslF6-V667M 3' fragment	GAGCGGCTCTTCCAGaTGCTCCGCTG	Wildtype *BdCslF6* Reverse

-

>BdCslF6|wildtype

PYRVLIFVRLIAFTLFVIWRISHKNPDTM-----WLWVTSICGEFWFGFSWLLDQLPKLNPINRIPDLAVLRQRFDRADG
TSTLPGLDIFVTTADPIKEPILSTANSVLSILAADYPVDRNTCYIS**DD**SGMHPYMGRAHDEFVNDRRRVRKEYDDFKAKI
NLVYMSREKRPGHNHQKKAGAMNALTRASALLSNAPFILNL**DCD**HYINNSQALRAGICFMVGRDSDTVAFVQFPQRFEGV
DPTD-------LYANHNRIFFDGTLRALDGMQGPIYVGTGCLFRRITVYGFGGWVYDTV**TED**VVTGYRMHIKGWRSRYCS
IYPHAFIGTAPINLTERLF**QVLRW**STGSLEIFFSKNNPLFGSTYLHPLQRVAYINITTYPFTAIFLIFYTTVPALSFVTG
HFIVQRPTTMFYVYLGIVLATLLIIAVLEVKWAGVTVFEWFRNGQFWMTASCSAYLAAVCQVLTKVIFRRDISFKLTSKL
PAGDEKKDPYADLYVVRWTPLMITPIIIIFVNIIGSAVAFAKVLDGEWTHWLKVAGGVFFNFWVLFHLYPFAKGLLGKHG
KT--

-----*

>*Bd*CslF6|A656T

PYRVLIFVRLIAFTLFVIWRISHKNPDTM-----WLWVTSICGEFWFGFSWLLDQLPKLNPINRIPDLAVLRQRFDRADG
TSTLPGLDIFVTTADPIKEPILSTANSVLSILAADYPVDRNTCYIS**DD**SGMHPYMGRAHDEFVNDRRRVRKEYDDFKAKI
NLVYMSREKRPGHNHQKKAGAMNALTRASALLSNAPFILNL**DCD**HYINNSQALRAGICFMVGRDSDTVAFVQFPQRFEGV
DPTD-------LYANHNRIFFDGTLRALDGMQGPIYVGTGCLFRRITVYGFGGWVYDTV**TED**VVTGYRMHIKGWRSRYCS
IYPHAFIGTTPINLTERLF**QVLRW**STGSLEIFFSKNNPLFGSTYLHPLQRVAYINITTYPFTAIFLIFYTTVPALSFVTG
HFIVQRPTTMFYVYLGIVLATLLIIAVLEVKWAGVTVFEWFRNGQFWMTASCSAYLAAVCQVLTKVIFRRDISFKLTSKL
PAGDEKKDPYADLYVVRWTPLMITPIIIIFVNIIGSAVAFAKVLDGEWTHWLKVAGGVFFNFWVLFHLYPFAKGLLGKHG
KT--

-----*

>RsBcsA|4P00

VVPVLLFLLWVALLVPFGLLAAAPVAPSAQGLIALSAVVLVALLKPFADKMV
PRFLLLSAASMLVMRYWFWRLFETLPPPALDASFLFALLLFAVETFSISIFFLNGFLSADPTDRPFP---------RPLQ
PEELPTVDILVPS---YNEPADMLSVTLAAAKNMIYPARLRTVVLC**DD**GGTDQRCMSPDPELAQKAQERRRELQQLCREL
GVVYSTRER----NEHAKAGNMSAALER----LKGELVVVF**DAD**HVP-SRDFLARTVGYFV--EDPDLFLVQTPHFFINP
DPIQRNLALGDRCPPENEMFYGKIHRGLDRWGGAFFCGSAAVLRRRALDEAGGFAGETI**TED**AETALEIHSRGWKSLYID
RAM--IAGLQPETFASFIQ**QRGRW**ATGMMQMLL-LKNPLF-RRGLGIAQRLCYLNSMSFWFFPLVRMMFLVAPLIYLFFG
IEIFVATFEEVLAYMPGYLAVSFLVQNALFARQRWP----LVSEVYEVAQ----APYLARAIVTTLLRPRSARFAVTAKD
ETLSE---NY-------ISPIYRPLLFTFLLCLSGVLATLVRWVAFP----GDRSVLLVVGGWAVLNVLLVGFALRAVAE
KQQRRAAPRVQMEVPAEAQIPAFGNRSLTATVLDASTSGVRLLVRLPGVGDPHPALEAGGLIQFQPKFPDAPQLERMVRG
RIRSARREGGTVMVGVIFEAGQPIAVRETVAYLIFGESAHWRTMREATMRPIGLLHGMARILWMAAASLPKTARDFMDEP
ARRRR*

Supplemental Figure 1. Protein sequences used to create an homology model of *Bd*CSLF6 and the A656T variant to the structure of BcsA from *R. sphaeroides* (PDB 4P00) (Morgan et al., 2013). HHpred alignments (Zimmermann et al., 2018) predicting homology were manually curated to define borders of the N-terminal, PCR and CSR domains of *Bd*CSLF6 which were excluded due to lack of homology to BcsA. In modelled regions, gaps where either low homology exists or where additional residues are present relative to BcsA are included with **dash** symbols. The D,D,D,QxxRW motif is shown in **bold**, native residue A656 is shown in **green** in the wildtype sequence, whilst the variant A656T is indicated in **red**.

```
                                                        CSR
                                                        ------>
AsCSLF6       QFPQRFEGVDPTDLYANHNRIFFDGSLRALDGMQGPIYVGTGCLFRRITVYAFDPPRINV
LmCSLF6       QFPQRFEGVDPTDLYANHNRIFFDGTLRALDGMQGPIYVGTGCLFRRITVYAFDPPRINV
HvCSLF6       QFPQRFEGVDPTDLYANHNRIFFDGTLRALDGMQGPIYVGTGCLFRRITVYGFDPPRINV
TaCSLF6       QFPQRFEGVDPTDLYANHNRIFFDGTLRALDGMQGPIYVGTGCLFRRITVYGFDPPRINV
BdCSLF6       QFPQRFEGVDPTDLYANHNRIFFDGTLRALDGMQGPIYVGTGCLFRRITVYGFDPPRINV
SbCSLF6       QFPQRFEGVDPTDLYANHNRIFFDGTLRALDGMQGPIYVGTGCMFRRITLYGFDPPRINV
ZmCSLF6       QFPQRFEGVDPTDLYANHNRIFFDGTLRALDGMQGPIYVGTGCLFRRITLYGFDPPRINV
OsCSLF6       QFPQRFEGVDPTDLYANHNRIFFDGTLRALDGLQGPIYVGTGCLFRRITLYGFEPPRINV
              *************************:******:**********:*****:*.*:******

                             Target region NGS screen

AsCSLF6       GGPCFPMLGGMFAKTKYQKPGLEMTMAKAK---AAPVP--AKGKHGFLPLPKKTYGKSDA
LmCSLF6       GGPCFPMLGGMFAKTKYEKPGLEMTMAKAK---AAPVP--AKGKHGFLPLPKKTYGKSEA
HvCSLF6       GGPCFPRLAGLFAKTKYEKPGLEMTTAKAK---AAPVP--AKGKHGFLPLPKKTYGKSDA
TaCSLF6       GGPCFPRLAGLFAKTKYEKPSLEMTMAKAK---AAPVP--AKGKHGFLPLPKKTYGKSDA
BdCSLF6       GGPCFPALGGLFAKTKYEKPSMEMTMARAN---QAVVPAMAKGKHGFLPLPKKTYGKSDK
SbCSLF6       GGPCFPSLGGMFAKTKYEKPGLELTT----------KAAVAKGKHGFLPLPKKSYGKSDA
ZmCSLF6       GGPCFPALGGMFAKAKYEKPGLELTTT---------KAAVAKGKHGFLPMPKKSYGKSDA
OsCSLF6       GGPCFPRLGGMFAKNRYQKPGFEMTKPGAKPVAPPPAATVAKGKHGFLPMPKKAYGKSDA
              ****** *.*:*** :*:**.:*:*            .  *********:***:****:
                                                 CSR
                                                 <------

                                    A600 G605     W614
AsCSLF6       FVDSIPLASHPSPYVAAYNTAEGIVTDEATMAEAVNVTAAAFEKKTGWGKEIGWVYDTVT
LmCSLF6       FVDSIPRASHPSP----YEPAETVATDDGIMAEAVNVTAAEFEKKTGWGKEIGWVYDTVT
HvCSLF6       FVDTIPRASHPSPY---AAAAEGIVADEATIVEAVNVTAAAFEKKTGWGKEIGWVYDTVT
TaCSLF6       FVDSIPRASHPSPY---AAAAEGIVADEATIVEAVNVTAAAFEKKTGWGKEIGWVYDTVT
BdCSLF6       FVDTIPRASHPSPY--AAEGIRVVDSGAETLAEAVKVTGSAFEQKTGWGSELGWVYDTVT
SbCSLF6       FVDTIPRASHPSPF-LSADEAAAIVADEAMITEAVEVCTAAYEKKTGWGSDIGWVYGTVT
ZmCSLF6       FADTIPMASHPSPF-AAAS-AASVVADEATIAEAVAVCAAAYEKKTGWGSDIGWVYGTVT
OsCSLF6       FADTIPRASHPSPY-AAEA---AVAADEAAIAEAVMVTAAAYEKKTGWGSDIGWVYGTVT
              *.*:** ******          : :.   :.*** *  : :*:**** *.::****.***

                                           A656       V667
AsCSLF6       EDVVTGYRMHIKGWRSRYCSIYPHAFIGTAPINLTERLFQVLRWSTGSLEIFFSKNNPLF
LmCSLF6       EDVVTGYRMHIKGWRSRYCSIYPHAFIGTAPINLTERLFQVLRWSTGSLEIFFSKNNPLF
HvCSLF6       EDVVTGYRMHIKGWRSRYCSIYPHAFIGTAPINLTERLFQVLRWSTGSLEIFFSKNNPLF
TaCSLF6       EDVVTGYRMHIKGWRSRYCSIYPHAFIGTAPINLTERLFQVLRWSTGSLEIFFSKNNPLF
BdCSLF6       EDVVTGYRMHIKGWRSRYCSIYPHAFIGTAPINLTERLFQVLRWSTGSLEIFFSKNNPLF
SbCSLF6       EDVVTGYRMHIKGWRSRYCSIYPHAFIGTAPINLTERLYQVLRWSTGSLEIFFSRNNPLF
ZmCSLF6       EDVVTGYRMHIKGWRSRYCSIYPHAFIGTAPINLTERLFQVLRWSTGSLEIFFSRNNPLF
OsCSLF6       EDVVTGYRMHIKGWRSRYCSIYPHAFIGTAPINLTERLFQVLRWSTGSLEIFFSRNNPLF
              **************************************:***************:*****

                     TMH3
                     ------>
AsCSLF6       GSTYLHPLQRIAYINITTYPFTAIFLIFYTT
LmCSLF6       GSTYLHPLQRVAYINITTYPFTAIFLIFYTT
HvCSLF6       GSTYLHPLQRVAYINITTYPFTAIFLIFYTT
TaCSLF6       GSTYLHPLQRVAYINITTYPFTAIFLIFYTT
BdCSLF6       GSTYLHPLQRVAYINITTYPFTAIFLIFYTT
SbCSLF6       GSTFLHPLQRVAYINITTYPFTALFLIFYTT
ZmCSLF6       GSTFLHPLQRVAYINITTYPFTAIFLIFYTT
OsCSLF6       GSTFLHPLQRVAYINITTYPFTALFLIFYTT
              ***:******:************:*******
```

Supplemental Figure 2. ClustalX2 alignment of CSLF6 protein sequences from various grasses including maize (Zm), rice (Os), barley (Hv), wheat (Ta), oat (As), *L. multiflorum* (Lm), *B. distachyon* (Bd) and sorghum (Sb). Sequence from the conserved structural motif, QxPx, through to the beginning of TMH3 is shown, a region which includes the target sequence for the NGS screen of the *B. distachyon* TILLING population indicated by **black line** above the sequences. Key catalytic motifs of the cellulose synthase domain in this region, xED and QxxRW, are marked in **bold**. The sequence of the CSR (Sethaphong et al., 2013; Dimitroff et al., 2016) is shown with **grey shading**, and the beginning of TMH3 indicated by a label above the sequence. The positions of the five amino acids where substitutions were identified, and that are predicted to be damaging, are shown with a **box** and the corresponding residue number above the sequence. Residues under selection in *HvCslF6* either within or predicted to interact with the CSR, I518, A612 and I643, are shaded in **pink** (Schwerdt et al., 2015).

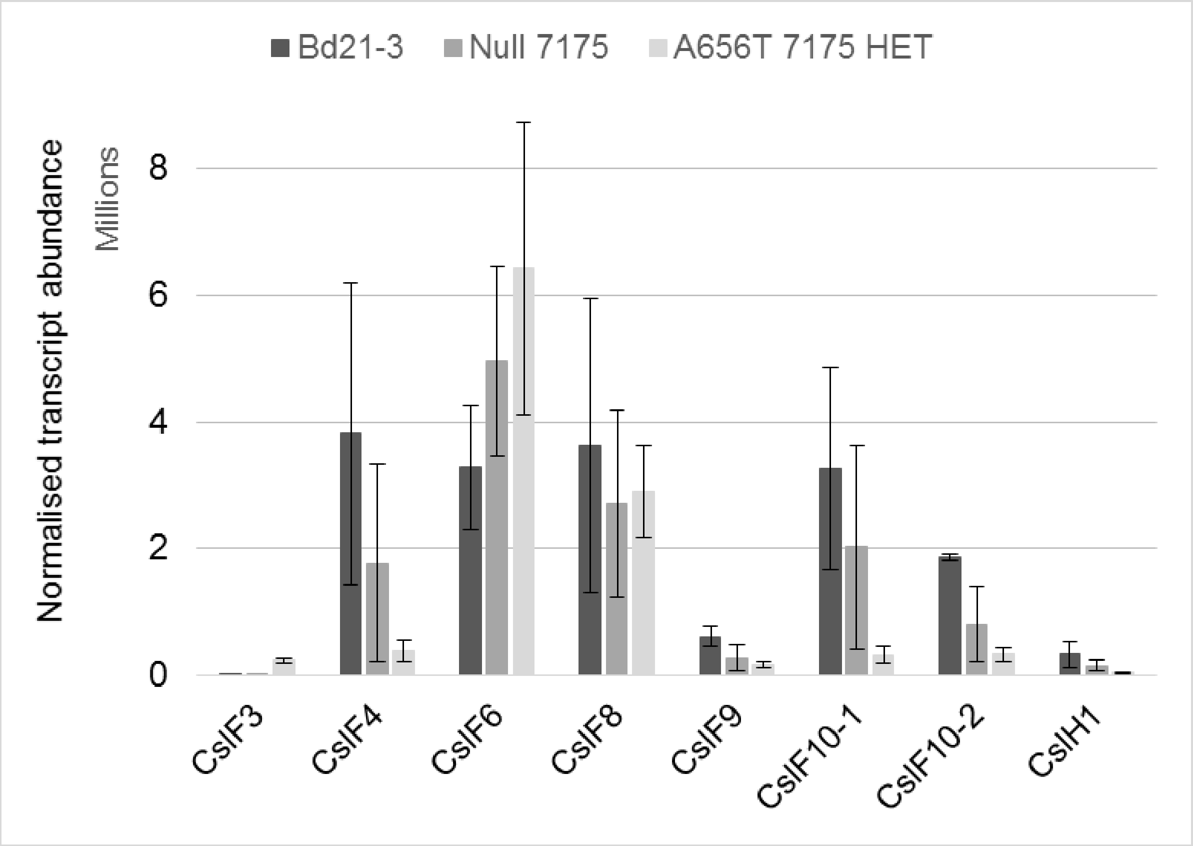

Supplemental Figure 3. Normalised transcript abundance of MLG synthase and related genes in *B. distachyon* grain 8 - 10 DAP. Expression has been normalised using the GeNORM method described by Vandesompele et al. (2002). Comparison of expression of *CslF* and *H* genes in Bd21-3 wildtype (n=6), Null 7175 (n=6) and A656T 7175 heterozygous individuals (n=4), shown as the average of replicate grain (+/- SE). The *CslF10-2* (Bradig25157.1) gene is a paralogue of *CslF10-1* (Bradig25150.1), as described by Ermawar et al. (2015).

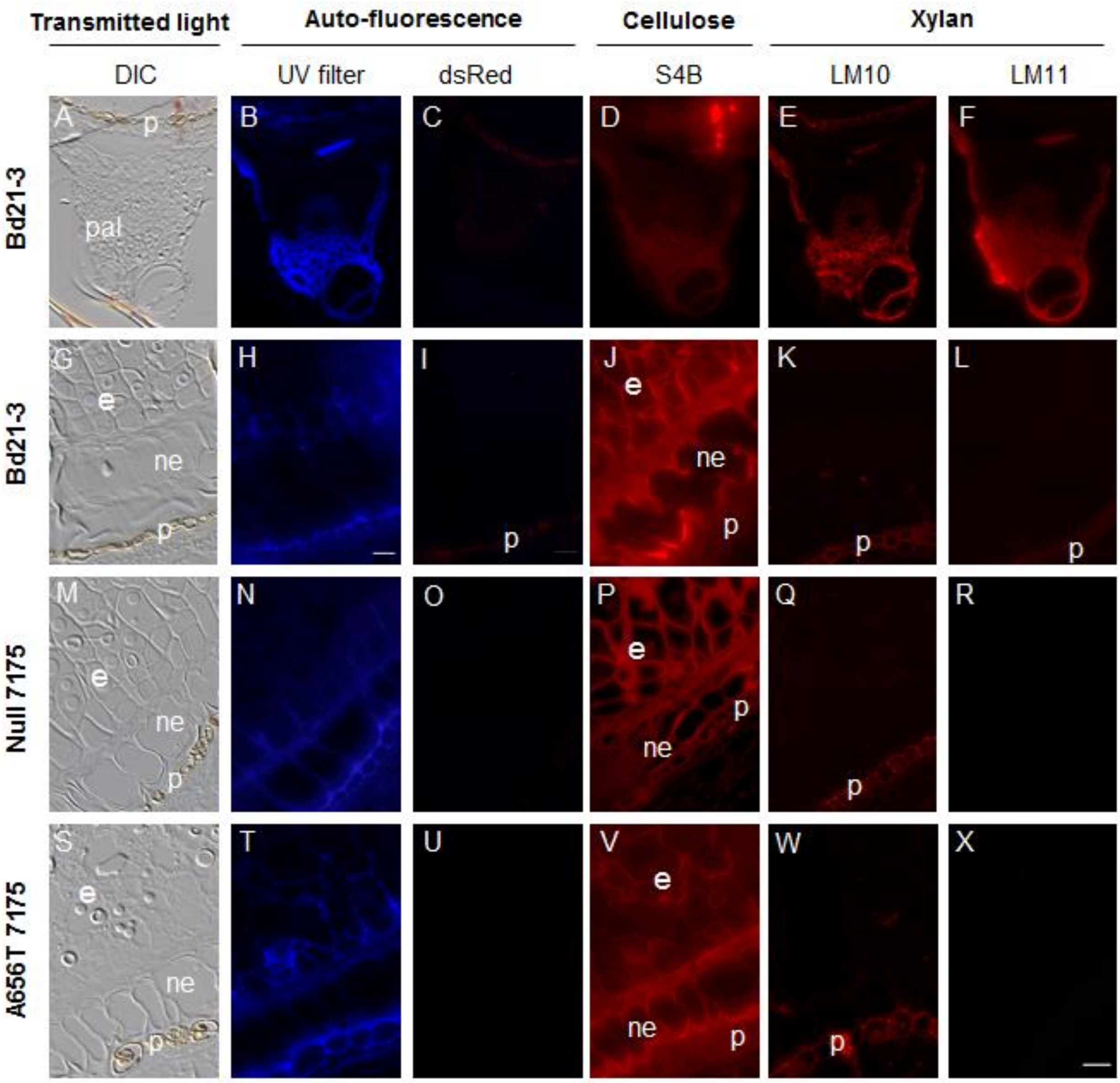

Supplemental Figure 4. Comparison of cellulose and xylan distribution in the cell walls of wild type Bd21-3 **(A-L)**, Null 7175 **(M-R)** and A656T 7175 **(S-X)** in grain 18-20 DAP. For wildtype grain detail of the palea **(pal; A-F)** is shown as a control for cellulose **(S4B)**, unbranched xylan **(LM10)** and branched xylan **(LM11)** labelling which is expected to be high. Labelling in endosperm **(e)**, nucellar epidermis **(ne)** and pericarp **(p)** is shown for all lines **(G-X)**. Strong labelling for cellulose was present in the endosperm, nucellar epidermis and pericarp with no observable difference between lines **(J,P,V)**. No labelling with either xylan antibody was observed in endosperm in any line, although some labelling of pericarp was observed with LM10 and sometimes LM11 **(K,L,Q,R,W,X)**. Scale bars show 20 µm.

References

Dimitroff, G., Little, A., Lahnstein, J., Schwerdt, J.G., Srivastava, V., Bulone, V., et al. (2016). (1,3;1,4)-β-Glucan Biosynthesis by the CSLF6 Enzyme: Position and Flexibility of Catalytic Residues Influence Product Fine Structure. *Biochemistry* 55(13)**,** 2054-2061. doi: 10.1021/acs.biochem.5b01384.

Ermawar, R.A., Collins, H.M., Byrt, C.S., Betts, N.S., Henderson, M., Shirley, N.J., et al. (2015). Distribution, structure and biosynthetic gene families of (1,3;1,4)-β-glucan in Sorghum bicolor. *Journal of Integrative Plant Biology* 57(4)**,** 429-445. doi: doi:10.1111/jipb.12338.

Morgan, J.L.W., Strumillo, J., and Zimmer, J. (2013). Crystallographic snapshot of cellulose synthesis and membrane translocation. *Nature* 493**,** 181-187. doi: 10.1038/nature11744.

Schwerdt, J.G., MacKenzie, K., Wright, F., Oehme, D., Wagner, J.M., Harvey, A.J., et al. (2015). Evolutionary Dynamics of the Cellulose Synthase Gene Superfamily in Grasses. *Plant Physiology Preview*. doi: 10.1104/pp.15.00140.

Sethaphong, L., Haigler, C.H., Kubicki, J.D., Zimmer, J., Bonetta, D., DeBolt, S., et al. (2013). Tertiary model of a plant cellulose synthase. *Proceedings of the National Academy of Science* 110(18)**,** 7512-7517. doi: 10.1073/pnas.1301027110.

Vandesompele, J., De Preter, K., Pattyn, F., Poppe, B., Van Roy, N., De Paepe, A., et al. (2002). Accurate normalization of real-time quantitative RT-PCR data by geometric averaging of multiple internal control genes. *Genome Biology* 3(7)**,** 1-12.

Wilson, S.M., Ho, Y.Y., Lampugnani, E.R., Van de Meene, A.M.L., Bain, M.P., Bacic, A., et al. (2015). Determining the Subcellular Location of Synthesis and Assembly of the Cell Wall Polysaccharide (1,3; 1,4)-β-d-Glucan in Grasses. *The Plant Cell* 27(3)**,** 754-771. doi: 10.1105/tpc.114.135970.

Zimmermann, L., Stephens, A., Nam, S.-Z., Rau, D., Kübler, J., Lozajic, M., et al. (2018). A Completely Reimplemented MPI Bioinformatics Toolkit with a New HHpred Server at its Core. *Journal of Molecular Biology* 430(15)**,** 2237-2243. doi: https://doi.org/10.1016/j.jmb.2017.12.007.
